# The Correlation between Leader–Member Exchange and Organisational Commitment among Spanish Registered Nurses: The Moderating Role of Sex and Hospital Size

**DOI:** 10.3390/ijerph17030721

**Published:** 2020-01-22

**Authors:** Nieves López-Ibort, Delia González-de la Cuesta, Teresa Antoñanzas-Lombarte, Ana Gascón-Catalán

**Affiliations:** 1 Miguel Servet University Hospital, 50009 Zaragoza, Spain; nlopezi@salud.aragon.es (N.L.-I.); dgonzalezd@salud.aragon.es (D.G.-d.l.C.); tantonnanzasl@salud.aragon.es (T.A.-L.); 2 Health Sciences Faculty, University of Zaragoza, 50009 Zaragoza, Spain

**Keywords:** nursing, work engagement, leadership, interpersonal relations, empowerment

## Abstract

The role of the supervisor in hospitals is to oversee and encourage the active work participation of registered nurses. In this context, leadership should be focused on the creation of a positive environment for the generation of high-quality care and the development of attitudes that have a beneficial influence on the work of the registered nurse. The aims of this study have been: (i) To verify if the quality of the supervisor–nurse interpersonal relationship was correlated with organisational commitment; (ii) to establish if the correlation could be moderated by empowerment, perceived organisational support, and leader–leader exchange. A cross-sectional survey with self-report questionnaires was performed. A total of 2541 registered nurses from nine public hospitals participated in the study. They completed scales measuring leader–member exchange, commitment, empowerment, perceived organisational support, and leader–leader exchange. There was a positive correlation between the quality of the leader–member exchange and commitment. Leader–leader exchange has a moderating effect on this relationship. The moderating effects of empowerment, perceived organisational support, and leader–member exchange on the supervisor–nurse interpersonal relationship and the nurse’s organisational commitment are influenced by sex and/or hospital size. Organisations should design supervisor training strategies aimed at establishing high-quality supervisor–nurse interpersonal relationships.

## 1. Introduction

Organisational commitment is a work attitude which concerns: The loyalty of the employee to the organisation; willingness to make an effort on behalf of the organisation; the degree of congruence of personal goals and organisational values; and the desire to remain a member of the organisation [1]. Organisational commitment has been positively correlated with participation in decision making, autonomy, working capacity, job satisfaction, and productivity [2]. Negative correlations have been reported with absenteeism and rotation [3,4]. Commitment is therefore a key indicator for the organisation, and it is instrumental in understanding other variables related to work and organisational outcomes such as job performance [5], organisational effectiveness, and work stress [6]. Organisational commitment is an attitude that all leaders should aim to instil in their subordinates. The loss of commitment is usually derived from the actions of the leader and/or the organisation [7].

In the case of hospitals, Kramer, Schmalenberg and Maguire [8] suggested that the supervisors embody the organisational leadership of the registered nurses. The supervisor has a significant influence on the work performance and the work attitudes of the nurse [9]. Previously published literature has consistently demonstrated that the exchange between the hierarchical superior and the subordinate (leader–member exchange) has a direct effect on organisational commitment [10]. The leader–member exchange has been identified as the factor that is most likely to improve commitment [11].

Despite the relatively extensive research that has been undertaken on the effects of the quality of the leader–subordinate relationship and work commitment, very few studies have been focused on the field of health care [12]. A recent study has been performed on nursing management and the quality of the supervisor–nurse relationship, paying special attention to its impact on nurses and work results [13]. A better understanding of quality of relationships between nurses and leaders can help hospital managers apply effective programmes to create a positive environment for the generation of constructive attitudes towards work. This research provides further empirical evidence about the supervisor–nurse relationship, as perceived by the nurse, with data reported by registered nurses who work in nine public hospitals (Spain).

### 1.1. LMX Theory

Leader–member exchange theory (LMX) differs from other leadership theories in that it emphasises the dyadic relationship between the leader and subordinate. It emerged as a critique of the approaches to leadership (dominant until the middle of the last century) which assumed that leaders treated all subordinates in the same way. The basic premise of LMX is the concept of differentiation [14]. The theory also postulates that the nature and quality of these relationships significantly affect the attitudes and behaviours of the leader and the subordinate [15]. The quality rating of the leader–subordinate relationship varies from high-quality relationships, characterised by extra-contractual behaviours, to low-quality relationships that are solely defined by contractual behaviours, hierarchy, and work roles [16]. In the development of these relationships, dimensions that are considered as “exchange currencies” are contribution, loyalty, affection, and respect [17]. This theory has served as the foundation for many articles over the last years [18].

### 1.2. LMX (m) and Organisational Commitment

Organisational commitment is an important management element that determines nurses’ work performance, productivity, and turnover intention [19,20]. Organisational commitment is influenced by work challenges, opportunities for social interaction, and feedback. Leaders are generally responsible for assigning tasks and providing feedback [21].

LMX (m) refers to the quality of the supervisor–nurse relationship, as perceived by the nurse. The quality of the LMX (m) is related to the amount of resources that employees receive from their leader and the perceived value of the exchange [22]. Employees that report a high-quality LMX (m) perceive: i) That their leaders trust them and give them more feedback [23]; ii) that they receive more challenging tasks [24] and obtain more leadership support in carrying our those tasks [16]; and iii) that the leaders offer high levels of support [25]. Moreover, close contact with the leaders means that interactions between leaders and subordinates become more frequent [26]. In contrast, low-quality LMX (m) relationships are exemplified by low levels of trust, limited support, and irregular feedback [23]. In line with the norm of reciprocity, high-quality leader–subordinate relationships can result in employees feeling a sense of obligation toward the organisation [27]. According to Dansereau, Graen, and Haga [28], subordinates in a high-quality LMX (m) receive both formal and informal benefits in exchange for their dedication and commitment to the organisation. Conversely, those who do not achieve high-quality relationships with leaders will probably feel that they are not offered the same formal and informal benefits as their colleagues (with a high-quality LMX) and their work commitment will be lower [29].

The first hypothesis of this study is as follows:

**Hypothesis** **1** *The quality of the supervisor–nurse relationship is positively correlated with the organisational commitment of the nurse.*

### 1.3. The Moderating Role of Empowerment in the Relationship between LMX (m) and Organisational Commitment

Empowerment has been shown to be a successful strategy for the promotion of positive work environments [30] and improved organisational results [31]. Following a literature review, Spreitzer [32] identified two dimensions of empowerment in the workplace: Structural empowerment (organisational structures and organisational processes that facilitate the optimal performance of employees) and psychological empowerment (employee responses to a specific work context). This approach sees psychological empowerment conceptualised through four constructs: Meaning; competence; self-determination; and impact.

One of the most important determinants of individual perception of empowerment is the quality of the relationship with the immediate superior [31]. Good relationships with superiors can have a variety of positive results for the employee: They are often given more responsibilities and resources; they feel empowered; work is valued as more meaningful; there is a greater perception of self-determination; and there is an improvement in competence [33]. Empowerment also contributes to organisational commitment through a process of reciprocity. Individuals are more likely to feel gratitude to organisations that give them more autonomy and more responsibilities and this makes the work more meaningful. Identification with the organisation and feelings of gratitude increase levels of commitment [34].

In short, there is clear empirical evidence that empowerment is positively correlated with organisational commitment [35]. If working conditions empower registered nurses for professional practice, supervisors should have access to more resources to distribute among the nurses to increase their commitment. Therefore, it is hypothesised that:

**Hypothesis** **2** *The empowerment of nurses enhances the positive correlation between their perceived quality of the leader–member exchange LMX (m) and organisational commitment: When there is more empowerment, the relationship between LMX (m) and commitment is stronger.*

### 1.4. The Moderating Role of Perceived Organisational Support (POS) in the Relationship between LMX (m) and Organisational Commitment

Perceived organisational support (POS) is the social exchange that refers to the global perceptions or beliefs that employees have on the extent to which organisations value their work contributions and care about their well-being [36]. Employees develop opinions and judgements on organisational support which have a significant effect on performance [37]. Employees perceive a high level of support when the organisation provides appropriate resources, and offers bonuses, rewards, and opportunities for advancement [38]. The relationship between positive work experiences and POS is stronger when the provision of these resources is attributed to the discretionary actions of the organisation, rather than decisions that are bound by external constraints [39].

The theory of social exchange [40] is the foundation for understanding the sense of obligation that is created with the organisation; when there is a high POS, workers develop confidence in their employer and make more effort to achieve the goals of the organisation [41]. In a similar manner, when employees perceive a lack of organisational support, the results are more likely to have a negative effect on the organisation; there may be increased absenteeism, reduced performance, and violations of standards [42]. Health care management practices that provide strong support for the professional practice of nursing lead to greater commitment to the organisation on the part of the nurse, either directly or indirectly [43]. Thus, the following hypothesis is proposed:

**Hypothesis** **3** *Perceived organisational support (POS) will enhance the positive relationship between the perceived quality of the leader–member exchange, LMX (m), and organisational commitment: When the POS is high/higher, the relationship between LMX (m) and commitment is stronger.*

### 1.5. The Moderating Role of Leader–Leader Exchange (LLX) in the Relationship between LMX (m) and Commitment

Leader–leader exchange (LLX) refers to the dyadic relationship between the supervisor and their immediate superior [42]. In the network of dyadic relationships, the ability of the supervisor to influence the work of their lower-level collaborators could be affected by the quality of the relationship they maintain with their immediate superior [44]. The LLX evaluates the quality of the relationship of the supervisor with their immediate superior. As with the LMX, a good quality supervisor–superior relationship means that the supervisor is able to gain access to better opportunities and resources. This, in turn, has an effect on the subordinate–supervisor relationship: The supervisor may be responsible for distributing resources among subordinates, but it should be remembered that it is the “supervisor’s ascendant line” that determines the quantity of resources that are made available [45]. A supervisor with a high-quality LLX may have more to offer subordinates with whom they also have a high-quality LMX (m), the positive relationship between LMX (m) and commitment is reinforced by the norm of reciprocity. Previous studies have found that higher quality LLX relationships strengthen the main effects of LMX quality on employees’ individual attitudinal outcomes [42,46].

**Hypothesis** **4** *The leader–leader exchange enhances the positive relationship between the nurses’ perceived quality of the leader-member exchange and their organisational commitment: When the LLX is high/higher, the relationship between LMX (m) and commitment is stronger.*

As mentioned above, previous LMX research has offered empirical evidence on the relationship between subordinates’ perceptions of the LMX relationship with their supervisor and organisational commitment. In addition, some LMX theorists have proposed that supervisor and subordinate characteristics such as gender, socio-economic status, age, and education influence the LMX relationship. Of these characteristics, the influence of gender has generated the most attention and impact regarding this relationship [47,48]. However, although the numerous studies have examined the moderating effects of gender on specific relationships between LMX and work attitudes such as organisational citizenship behaviour [49], to date, there is no comprehensive published research that discusses the moderating effect of sex on the relationship between LMX and organisational commitment. A larger work group size and greater workload have been found to have direct negative effects on employee satisfaction and commitment [50]. Therefore, it seems plausible that gender and hospital size could moderate the relationship between subordinates’ perceptions of the quality of the LMX with their supervisor and their organisational commitment.

As previously mentioned, there appear to be no studies that have examined the effect of sex and hospital size on the relationship between LMX (m) and organisational commitment, given the large variations in the size of hospitals and the fact that the majority of registered nurses are women, the final hypothesis of this present work is:

**Hypothesis** **5** *Hospital size and the sex of the nurse influence the moderating effect of empowerment, POS, and LLX in the relationship between nurses’ perceived quality of the LMX (m) and their organisational commitment.*

### 1.6. Aims

**Figure 1:**
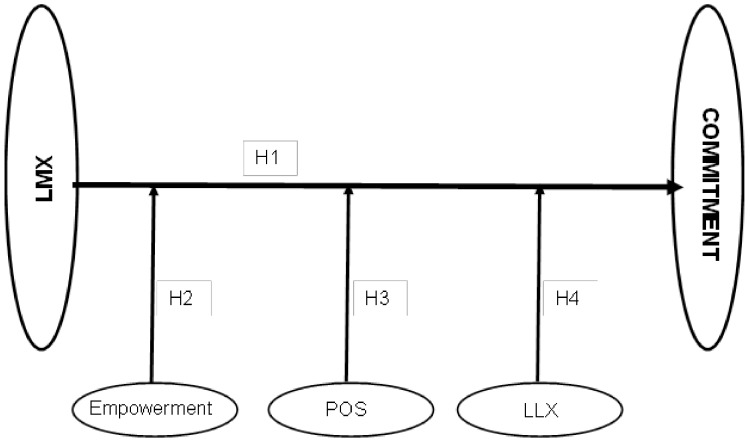
The research framework.

This research has three main aims:

(a) To verify the supposition that the quality of the supervisor–nurse interpersonal relationship correlates with organisational commitment;
(b) To establish if the variables empowerment, perceived organisational support, and leader–leader exchange act as moderators in this relationship (Figure 1);
(c) To determine if the moderating effect of these variables is influenced by the size of the hospital and the sex of the nurse.

## 2. Materials and Methods

### 2.1. Design

A descriptive, cross-sectional study was performed among Spanish registered nurses working in public hospitals. Anonymous questionnaires measuring the quality of the leader member exchange between nurses and supervisors, commitment, empowerment, perceived organizational support, and leader–leader exchange were self-administered in all wards of the hospitals that were included in the study. Demographic variables were also collected. The research unit was the nurse–supervisor dyad.

### 2.2. Sample/Participants

The universal population comprised all registered nurses and their supervisors who were working at the time of the study in the 9 general public hospitals in the Regional Community of Aragon (Spain). To be included in the study, the nurse must have been working with the same supervisor for at least one month. The one-month criterion is based on the work of Liden, Sparrowe, and Wayne [51] who found that leader–subordinate relationships tend to be established in a minimum period of two weeks. The total number of registered nurses was 4756. Of these, 3628 fulfilled the inclusion criteria and received questionnaires. A total of 2724 nurses returned questionnaires; 183 were incomplete and were rejected. The non-participation rate of nurses that met the study inclusion criteria was 29.96% (1087 nurses), and 44.4% (2115 nurses) in relation to the universal population. Hospital size ranged from 122 to 1290 beds.

### 2.3. Data Collection

The research team leader contacted the management of the hospitals in order to explain the project and to request their permission to approach the nursing staff. The nursing directors (or their representatives) were asked to organise a meeting with supervisors to discuss the study and invite collaboration. Subsequent meetings were held with principal researchers, the ward supervisors, and one or two registered nurses from all wards of the nine hospitals. Contact details were facilitated in case there were any problems when completing the questionnaire.

The questionnaire and an informative leaflet were distributed to the participants in an envelope that could be sealed on completion and return in order to ensure confidentiality. Participation was voluntary; the questionnaires were distributed, collected, and returned to the research team by the registered nurses who had attended the meetings. The questionnaires were identified with the number of the hospital and the nursing care ward. Data was collected between April and June 2016.

### 2.4. Measures

The measurement instruments were selected after an extensive review of the literature. Two criteria were considered: Content validity and the frequency with which the instrument had been used in previous studies.

#### 2.4.1. Leader–Member Exchange

The perceived quality of the relationship with the supervisor was measured by the one-dimensional adapted questionnaire LMX-7 (leader member exchange), developed by Graen and Uhl-Bien [52]. The questionnaire has 7 items and a Líkert scale with 5 response options from 1 (rarely) to 5 (very often). It was validated in the Spanish language by De la Rosa and Carmona [53]. The Cronbach alpha was 0.925.

#### 2.4.2. Organisational Commitment

Organisational commitment was evaluated using an adaptation of the 9-item short version of the OCQ (Organisational Commitment Questionnaire) developed by Mowday, Steers, and Porter [54]. The questionnaire measures the desire to remain in the organisation, the maintenance of high levels of effort, and the acceptance of organisational goals and values. Items are scored on a Líkert scale with 7 response options ranging from 1 (strongly disagree) to 7 (strongly agree). It was validated in the Spanish language by De la Rosa and Carmona (2010). The Cronbach alpha was 0.894.

#### 2.4.3. Empowerment

The perception of empowerment was measured by an adapted version of the 13-item Spreitzer questionnaire [55]. The instrument measures autonomy, competence, impact, and meaning. There are three items for each of the four dimensions of empowerment, with the exception of autonomy that has four. Items are scored on a 5-point Likert scale ranging from 1 (a little) to 5 (a lot). The Cronbach alpha was 0.881.

#### 2.4.4. Perceived Organisational Support

POS was measured by a seventeen-item abbreviated version of the Survey of Perceived Organisational Support [36]. The questionnaire was validated in the Spanish language by Ortega [56]. Items are evaluated on a 7-point Likert scale ranging from 1 (strongly disagree) to 7 (strongly agree). The Cronbach alpha was 0.938.

#### 2.4.5. Leader–Leader Exchange

As previously mentioned, with the exception of the different positions of those who make up the dyadic relationship, the LLX is basically the same as the LMX; the supervisor’s satisfaction with the quality of the relationship with their immediate superior was therefore measured in the same way as the LMX (m), using the one-dimensional adapted LMX-7 developed by Graen and Uhl-Bien [52]. The Cronbach alpha was 0.947.

### 2.5. Demographic Variables

The questionnaire included socio-demographic variables: Age; sex (male/female); time working as a nurse; time working in the current hospital; time working in the current unit; time working with the current supervisor; working hours (full/part-time); and hospital size—large (more than 501 beds) or small (500 beds or less).

### 2.6. Ethical Considerations

Data confidentiality and anonymity were guaranteed. An envelope was included with the questionnaires which was sealed and returned on completion. An individual code was assigned to each questionnaire so the participant could not be identified at any time.

The project was approved by the Clinical Research Ethics Committee of Aragón (C.I. PI16 / 0106).

### 2.7. Data Analysis

The statistical analysis was performed with SPSS version 22.0 (IBM Corp., Armonk, NY, USA). Data was analysed using descriptive and inferential statistical techniques.

The analysis comprised five stages:

1. The descriptive statistics were calculated.
2. An exploratory factor analysis was carried out for each variable to analyse the construct validity of the scales. The Kaiser–Meyer–Olkin test was utilised to check the suitability of the sampling for the factor analysis (values close to 1). The Bartlett sphericity was implemented to check that the extraction of factors was adequate and that the factorial analysis was significant for all cases. The Main Components method for the extraction of factors was also used and an orthogonal rotation was undertaken with the Varimax method. In order to verify the consistency of the instruments used to measure the variables the Cronbach alpha test was employed with each scale. The reliability values were very high, indicating good levels of consistency.
3. A correlation matrix gave further variable crossings and a regression model forecast the LMX (m) based on the commitment of the nurse. The regression model followed the Stepwise methodology which allows for controlling inter-correlations among independent variables.
4. A multiple linear regression model was used to check the intervention of moderating variables. The equation included the dependent variable (Y), the independent variable (X), the moderating variable (Z), and the product of the independent moderator (X × Z). The significance of the latter term indicates whether the variable in question is a moderating variable.
5. The estimations were repeated by sub-samples of sex (men/women) and hospital size (large/small) in order to verify the weighting of these variables in the study.

## 3. Results

### 3.1. Descriptive Statistics

A total of 3628 questionnaires were given to the registered nurses; 2724 were returned and 2541 were suitable for the analysis, a response rate of 70.04%.

Most participants (91.3%) were women (n = 2319). The average age was 44 years (SD = 11). Average work experience was 19.6 years (SD = 11.3); average time working in the current hospital was 14.5 years (SD = 11.9); average time in the current unit was 8.2 years (SD = 9.9); and average time working with the current supervisor was 3.9 years (SD = 5.4). A large majority of the registered nurses worked full-time (78.4%).

Almost two thirds of the registered nurses (64.1%) worked in the two large hospitals of the region; the remainder (35.9%) were employed in the seven smaller hospitals.

### 3.2. Hypotheses

#### 3.2.1. Hypothesis 1

The results confirmed a positive correlation between the LMX (m) and commitment (r = 0.232, *p* < 0.01). The positive coefficient sign indicates that the higher the perceived quality of the relationship, the greater the commitment.

A multiple linear regression model was used to test the moderating variables.

The initial equation of the commitment of the nurse and the independent variable LMX (m) is:

Commitment = 2.92 + 0.45 x LMX (m) + e
The percentage of explained variance (R^2^) is 11.4%.

#### 3.2.2. Hypothesis 2

Commitment = 1.63 + 0.19 x LMX (m) + 0.48 x Empowerment – 0.04 x (LMX (m) x Empowerment) + e.

With a variance percentage of 19.4% and a non-significant product coefficient (t = 0.86 and *p* > 0.05), there was no empirical evidence that empowerment acts as a moderator in the relationship between LMX (m) and commitment. The gap between the initial variance and the variance with empowerment as the moderating variable was 70%.

#### 3.2.3. Hypothesis 3

Commitment = 2.25 + 0.10 x LMX (m) + 0.47 x POS + 0.03 x (LMX (m) x POS) + e.

With a variance percentage of 35.1% and a non-significant product coefficient (t = 1.58 and *p* > 0.05), there was no empirical evidence that perceived organisational support acts as a moderator in the relationship between LMX (m) and commitment. The gap between the initial variance and the variance with perceived organisational support as the moderating variable was 208%.

#### 3.2.4. Hypothesis 4

Commitment = 2.64 + 0.58 x LMX (m) + 0.05 x LLX – 0.03 x (LMX (m) x LLX) + e.

The variance percentage increased to 11.8% and the coefficient of the product variable was significant (t = −3.16 and *p* < 0.01). Therefore, the empirical evidence shows that the LLX exerts a moderating effect on the relationship between the LMX (m) and commitment. The gap between the initial variance and the variance with LLX as the moderating variable was 4%

#### 3.2.5. Hypothesis 5

A multiple linear regression model was used to check if the moderating effect of the variables in the relationship between the quality of LMX (m) and commitment depends on the nurse’s sex and hospital size.

Previous steps carried out for the total sample were repeated for sub-samples of sex and hospital size. The variance percentages are shown in Table 1.

Table 2 shows the initial, moderating coefficients for the commitment variable by nurse’s sex and hospital size. It can be seen that the relationship between LMX (m) and commitment is significant and that the coefficient is higher for women than for men. This relationship is only moderated by the LLX variable (and in a negative way); when stratifying by sex the effect was only observed for women. The moderating effect of empowerment on the LMX (m)-commitment relationship was significant for men.

**Figure 2:**
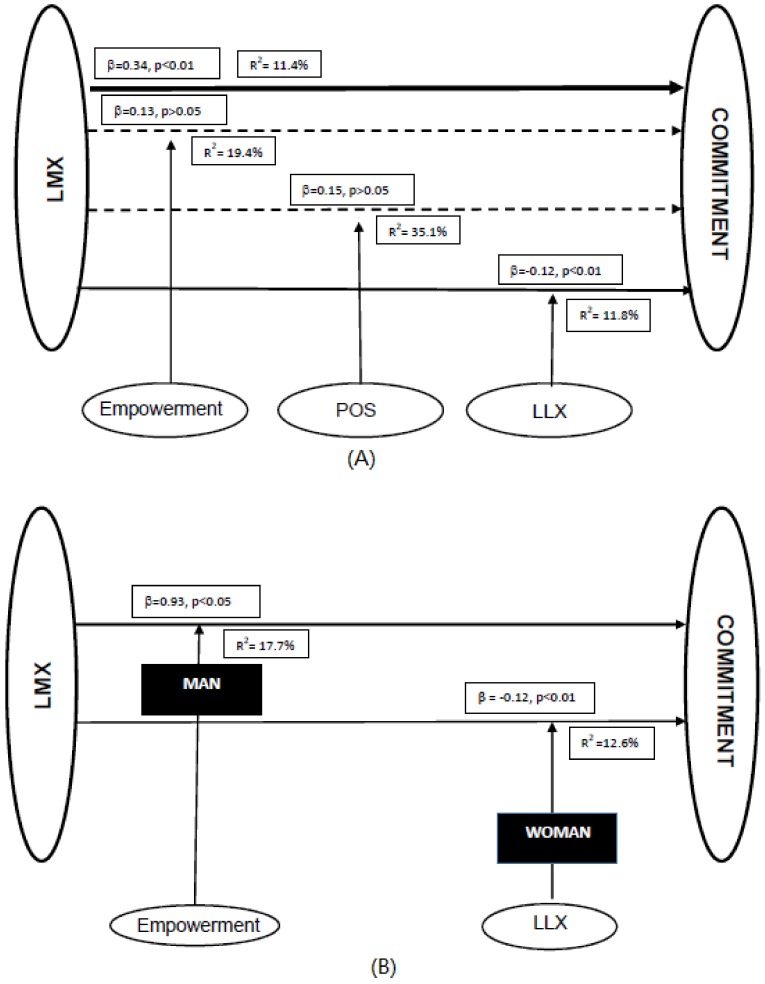

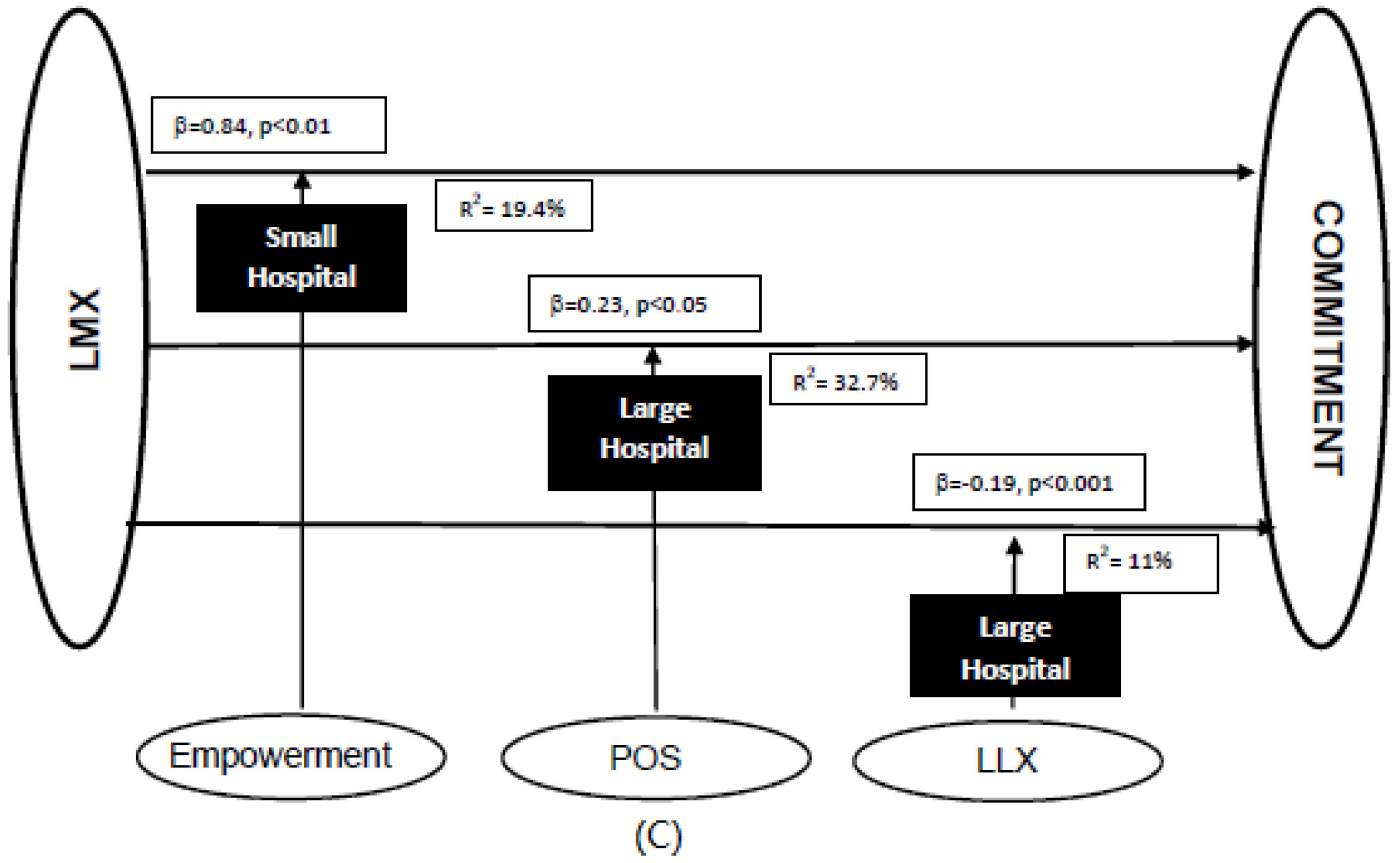
The hypothesised model with standardised parameters. (**A**): The moderating effect of the variables: Empowerment, perceived organisational support (POS), and leader–leader exchange (LLX) on the LMX-commitment relationship (dotted line indicates no effect). (**B** and **C**): The influence of sex and hospital size on the moderation of the variables in the LMX-commitment relationship. In B and C, only statistically significant associations are represented.

The effect of the moderation of the LLX and perceived organisational support when disaggregated by hospital size was only significant for large hospitals. The empowerment variable moderates this relationship for small hospitals. Figure 2 shows the hypothesised model.

## 4. Discussion

The results of this study confirm the findings of much of the previously published literature on the issue: The quality of the LMX (m) is positively correlated with organisational commitment [18]. However, it should be noted that not all research has concluded that this relationship is so clear and direct [57].

In the specific case of nurses, studies have also found that there is a positive relationship between LMX (m) and the level of affective commitment and increased feelings of emotional attachment to the hospital [58,59]. This result suggests that leadership encourages the collaborators’ commitment and empowerment, thus leadership also leads to higher standards of work and organisational outcomes [60], and turnover intention [19]. Consequently, leadership practices may have important positive implications for nursing staff and patient outcomes. Supervisors should be made aware of the importance of developing high-quality relationships with registered nurses. In addition, organisations should plan interventions aimed at empowering supervisors and enabling them to establish high-quality LMX relationships.

Previous studies have focused on the moderating role of LMX [61], however less research has been carried out on the moderating effects of other variables on the relationship between the LMX and work outcomes. There is scant literature that deals with moderating effects and the same variables that have been analysed in the present work.

The importance of developing and nurturing empowerment as a means to proactively address the changing challenges in the health sector is widely recognised [62]. Previous research suggests that the greater the nurse’s perception of empowerment, the higher is the level of affective commitment, loyalty, and emotional attachment to the hospital [60,63,64,65,66]. Nevertheless, the results of the present work did not confirm the moderating effect of empowerment in the relationship between LMX (m) and commitment when the whole sample was analysed without stratification. As far as the authors are aware, the present study is the first to consider sex and hospital size as moderators; results confirm that there are moderating effects regarding men and small hospitals but not for women and large hospitals.

When empowerment is introduced as a variable, the explained variance of the commitment and the LMX (m) almost doubles, from 11.4% to 19.4%. This finding could imply that the variable has another type of effect on the relationship, for example, a mediation effect.

Perceived organisational support (POS) was also considered as a moderating variable. The theory of organisational support argues that, in line with the reciprocity norm, employees respond to favourable treatment with greater organisational commitment [36]. The positive relationship between POS and organisational commitment is well established [67]. Furthermore, it has been substantiated in the field of health care [43] and among nurses [59,68].

The results of the present study did not confirm that the POS has a moderating effect on the relationship between LMX (m) and organisational commitment when the whole sample was analysed without stratification. Sample stratification by sex also gave no significant results. However, POS does seem to have a moderating effect in large hospitals. When POS is introduced as a moderating variable, the explained variance of the commitment and the LMX (m) rises from 11.4% to 35.1%, an increase in variance of more than 200%. Therefore, the POS appears to exert some other, non-moderating effect.

Finally, a slight moderating effect of the LLX in the main relationship between the LMX (m) and organisational commitment was observed. Stratification by nurse’s sex and hospital size revealed that the moderating effect of the LLX was significant for women and large hospitals. The lack of previous empirical evidence makes discussion on the robustness of this result impossible.

The results of the present work indicate that more research should be undertaken on how different contexts (e.g., hospital size) or the characteristics of the nurses could affect behaviour variables at work, the health results of the organisation, and patient care.

This study shows the importance of implementing management practices that promote high-quality supervisor–nurse relationships. Hospital management should not be limited to overseeing the performance of the supervisor; it should also consider the supervisor’s relationship with the nurse. The supervisor should be encouraged to develop a leadership style that prioritises the establishment of high-quality interpersonal relationships. Training should be used to raise awareness of the influence that the supervisor has on the nurses’ work attitudes and interpersonal skills that engender support, respect, and trust.

### Limitations

The robustness of cross-sectional research inferences is often hindered by problems of causality. Self-reported questionnaires tend to focus on some of the dimensions of the phenomenon and can fail to contemplate contextual variables [69]. Self-reporting may also cause common method variance [70] and this may affect the strength of the association among the variables.

Further limitations are: (a) The responses of the nurses may have been influenced by the principle of social desirability; (b) although anonymity was guaranteed, the nurses could have feared identification; (c) the study was based on nine public hospitals, no private organisations were included; and (d) there are other variables (procedural justice, range of control, implicit theories of leadership, the organisational culture, differentiation in the intra-group LMX, etc.) that could influence the model, it would be interesting to include them in future works.

## 5. Conclusions

This research confirms that the quality of the relationship that the supervisor establishes with the registered nurse is antecedent to the nurse’s organisational commitment. As this commitment is positively correlated with behaviour that benefits the organisation, there are clear implications that strategies that strengthen these attitudes should be designed and implemented. The strategies should be aimed at the development of training programmes and solutions so that supervisors and managers can foster their interpersonal relationship skills. It might also be valuable to include these programmes in hospitals or universities as specific training for students and/or professionals who are interested in supervisory or managerial positions.

It would also be useful to study and analyse efficacy of such programmes and to evaluate the interventions that are the most effective in terms of the improvement of supervisor–nurse relationships and to confirm the positive effect on organisational commitment.

Given the importance of the supervisor–nurse dyadic, it could be a good idea to evaluate supervisors in terms of the LMX. This would mean that quality of the supervisor–nurse relationship would become an indicator of performance, giving it the same importance as other, currently available, clinical indicators.

Due to the fact that the influence of empowerment and perceived organisational support on the behaviour of the nurse cannot exclusively be explained by a moderating effect, more research on other effects such as covariance, spuriousness, and mediation is required. As sex and/or hospital size may influence the moderating effect of empowerment, POS, and LLX in the relationship between the LMX (m) and organisational commitment should be included in the analysis of contexts and personal characteristics of the workers in future research and this could be used as a framework for high-level health management decisions.

Finally, it should be noted that most empirical evidence on this question refers to men working in private companies in North America and Anglo-Saxon countries. This study contributes to the growing literature on leadership and organisational commitment among nurses in a Spanish context. The work was based on nursing ward supervisors and it would be interesting to undertake research to test its applicability to other managerial areas or health professionals.

## Figures and Tables

**Table 1:** Explained variance percentages for the leader–member exchange theory (LMX)-commitment relationship with the initial model and after the three moderating variables; global and categorised values by sex and hospital size.

		Initial	LLX	POS	EMP
**Sex**	Men	5.1	6.7	33.6	17.7
	Women	12.1	12.6	35.4	19.7
**Hospital size**	Small	13.4	14.5	34.9	19.4
	Large	9.6	11.0	32.7	19.1
**Global**		11.4	11.8	35.1	19.4

**Table 2:** Regression coefficients (initial and after three moderations) for the LMX-commitment relationship; global and stratified values by sex and hospital size.

		Initial	LLX	POS	EMP
**Sex**	Men	0.284 ***	−0.051	0.113	0.240 *
	Women	0.466 ***	−0.028 **	0.024	0.005
**Hospital size**	Small	0.495 ***	0.005	−0.015	0.222 **
	Large	0.403 ***	−0.047 ***	0.051 *	−0.036
**Global**		0.451 ***	−0.028 **	0.032	0.035

* *p* < 0.05; ** *p* < 0.01; *** *p* < 0.001.
